# Rapid bedside multimodal ultrasound screening for acute type A aortic dissection: a perspective

**DOI:** 10.3389/fcvm.2026.1780067

**Published:** 2026-04-01

**Authors:** Hua Xu, Jin-Yan Yang

**Affiliations:** Department of Ultrasound, Shaanxi Provincial Hospital of Chinese Medicine, Xi’an, China

**Keywords:** acute type a aortic dissection, cardiac ultrasound, clinical decision-making, emergency medicine, multimodal point-of-care ultrasound, rapid screening

## Abstract

Acute type A aortic dissection (ATAAD) is a life-threatening cardiovascular emergency where delays in diagnosis worsen outcomes. While computed tomography angiography remains the diagnostic gold standard, its requirement for patient transport, contrast administration, and radiation exposure limits its utility in urgent scenarios. Multimodal point-of-care ultrasound (PoCUS) presents a viable bedside alternative by combining transthoracic echocardiography, vascular ultrasound, and, when available, transesophageal echocardiography. This integrated approach facilitates a rapid, comprehensive assessment, allowing for the detection of key findings—such as pericardial effusion, aortic root dilation, and an intimal flap—within minutes. PoCUS can shorten the time from clinical suspicion to treatment decision, avoid the risks associated with transporting unstable patients, and assist in differentiating other causes of acute chest pain. This perspective article scrutinizes the role of multimodal PoCUS in screening for ATAAD, addresses its technical and training limitations, and explores future directions, including integration with artificial intelligence, standardized training protocols, and optimized clinical workflows. The goal is to inform the development of more efficient, timely diagnostic strategies for this critical condition.

## 1 Introduction

Acute type A aortic dissection (ATAAD) is a lethal cardiovascular emergency. Its pathophysiology originates from a tear in the aortic intima, enabling blood to enter and propagate within the vessel wall, forming a false lumen (1, 2). This dissection can rapidly progress to aortic rupture. Mortality increases by approximately 1%–2% per hour following symptom onset (1, 3), and in-hospital death rates remain substantial despite therapeutic advances (1). Timely diagnosis is paramount for survival, as diagnostic delays directly impede emergent surgical intervention, exposing patients to the risk of fatal complications such as aortic rupture or malperfusion-related organ failure before reaching the operating room (2–4).

It is essential to recognize, however, that acute aortic dissection often presents with variable and sometimes atypical clinical features (5). While classic symptoms such as sudden, tearing chest or back pain are frequently highlighted, many patients exhibit non-specific manifestations, including syncope, abdominal pain, stroke-like neurological deficits, or isolated hypotension (6). These ambiguous presentations can obscure the underlying diagnosis and contribute to delays in recognition, particularly in emergency settings (7). Overreliance on classical symptomatology may therefore result in missed or delayed diagnoses, compounding the time-sensitive nature of this condition (8).

Computed tomography angiography (CTA) is the gold-standard modality for confirming ATAAD, providing high-resolution anatomical detail (9). However, its application in critically unstable patients is constrained by significant limitations (10). Transporting these patients to the scanner is inherently hazardous. Furthermore, the requisite iodinated contrast media carries a risk of nephrotoxicity (11), and the procedure involves exposure to ionizing radiation (11). These factors can introduce dangerous delays or contraindicate CTA use in emergencies, creating a critical diagnostic gap for high-risk individuals (12).

Point-of-care ultrasound (PoCUS) presents a viable bedside alternative, enabling rapid, non-invasive assessment without patient transfer (13, 14). Given the anatomical complexity of ATAAD, a systematic, multimodal PoCUS protocol is advocated (15). This involves the targeted integration of focused cardiac ultrasound (to evaluate the aortic root and pericardium), abdominal aortic ultrasound, and vascular assessments (e.g., carotid or femoral arteries) based on clinical presentation (16, 17). This tailored approach facilitates the prompt acquisition of key diagnostic findings to guide immediate clinical decision-making.

Although multimodal PoCUS holds potential for the early screening of ATAAD, its precise diagnostic role and integration into clinical practice require further clarification (18). Persistent challenges concerning diagnostic accuracy, operator training, protocol standardization, and workflow integration must be resolved (19). This perspective article aims to scrutiny the current evidence for multimodal PoCUS in ATAAD screening, analyze its associated technical and operational challenges, and discuss future directions for development, training, and implementation. The overarching objective is to explore how this approach may help mitigate diagnostic delays and improve outcomes in this time-sensitive emergency.

## 2 Core applications and value of multimodal PoCUS in ATAAD screening

Multimodal PoCUS provides a structured framework for the rapid bedside screening of suspected ATAAD. By sequentially integrating targeted ultrasound examinations, this approach generates critical diagnostic information to guide urgent clinical decisions.

### 2.1 A goal-oriented multimodal screening pathway

**Figure 1:**
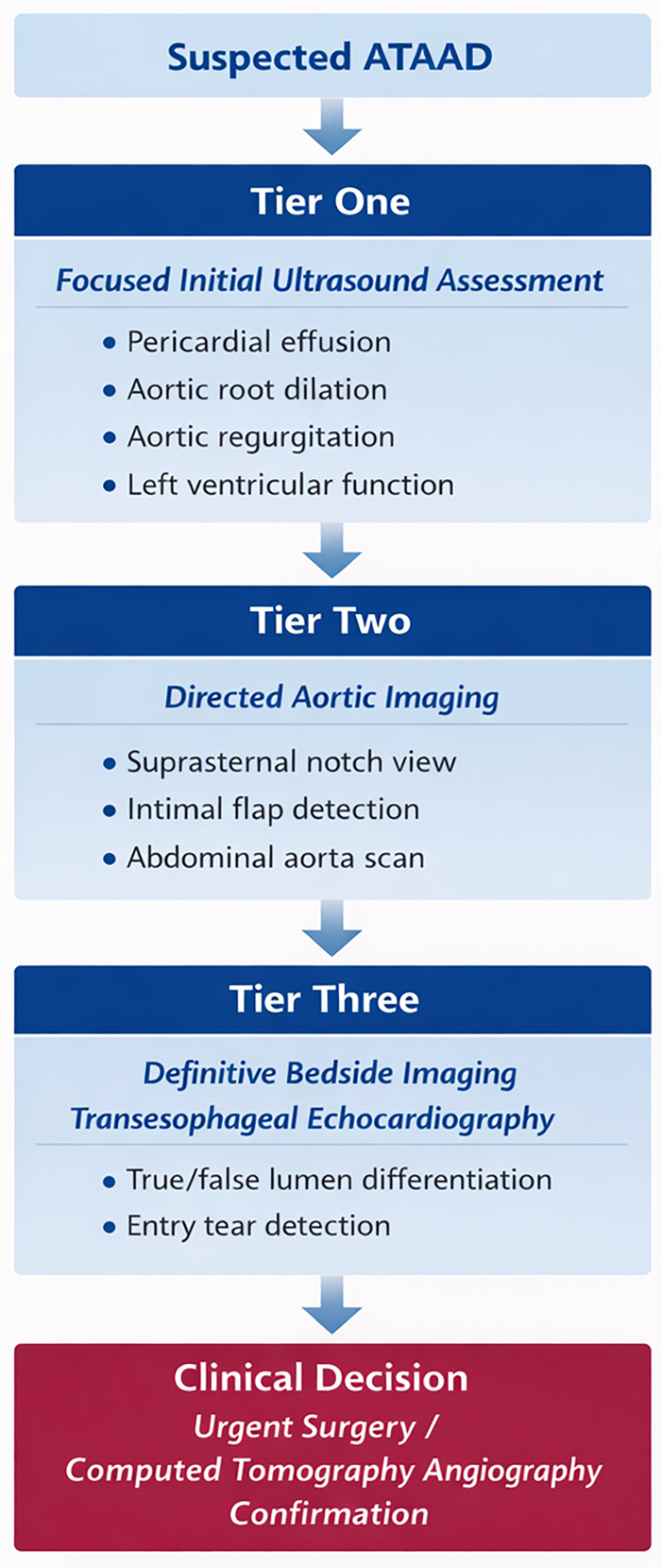
Tier-Based multimodal PoCUS screening pathway for suspected acute type A aortic dissection (ATAAD).

The screening strategy employs a three-tiered, stepwise logic designed to efficiently progress from initial screening toward diagnostic confirmation (Table 1). To enhance conceptual clarity, the screening framework is presented as a three-tiered diagnostic pathway (Figure 1). Each tier represents a progressively focused stage in the ultrasound evaluation process. The pathway begins with the rapid identification of indirect signs of aortic dissection and advances toward the direct visualization of the underlying aortic pathology. This structured, tier-based approach supports swift clinical decision-making while remaining consistent with established diagnostic protocols.

**Table 1:** Tier-based multimodal PoCUS screening strategy for suspected ATAAD.

Screening tier	PoCUS modality	Key targets	Suggestive findings	Immediate clinical implication
Tier 1	TTE	Pericardium, aortic root, aortic valve	Effusion, root dilation, AR	Identify life-threatening complications
Tier 2	Suprasternal/abdominal US	Arch, descending aorta	Intimal flap, lumen abnormality	Increase diagnostic certainty
Tier 3	TEE	Thoracic aorta	Flap, true/false lumen	Guide definitive decision

ATAAD, acute type A aortic dissection; PoCUS, point-of-care ultrasound; TTE, transthoracic echocardiography; TEE, transesophageal echocardiography; US, ultrasound; AR, aortic regurgitation; CTA, computed tomography angiography.

#### Tier one: focused initial ultrasound assessment

The primary objective is the rapid detection of life-threatening complications or indirect signs suggestive of ATAAD (Table 1). The examination focuses on four key domains (17). First, the pericardium is assessed for effusion, with specific attention to signs of tamponade such as right heart chamber collapse (20). Second, the aortic root diameter is measured, as significant dilation is a cardinal warning sign (21). Third, the aortic valve is evaluated for acute regurgitation (17). Finally, left ventricular function is assessed to help exclude alternative diagnoses (e.g., myocardial infarction) and guide resuscitation (17). A negative initial screen does not rule out ATAAD but can identify patients requiring immediate intervention for associated complications.

#### Tier two: directed aortic imaging

If clinical suspicion persists, this tier involves direct sonographic evaluation of the aorta using several critical transthoracic imaging windows. In addition to the suprasternal notch view, the parasternal long-axis view represents a key transthoracic window for evaluating the proximal ascending aorta and measuring the aortic root diameter (22, 23). This view enables clinicians to identify abnormal dilation of the ascending aorta and may occasionally reveal an intimal flap extending into the proximal aortic segment (24). The suprasternal notch view targets the aortic arch and proximal ascending aorta to identify a direct sign of dissection: an undulating intimal flap within the aortic lumen (25, 26). A concurrent abdominal aortic scan assesses for distal extension of the dissection and evaluates flow to major visceral arteries (27). A positive finding at this stage significantly raises the diagnostic probability of ATAAD. Collectively, these directed examinations expand the diagnostic field beyond the initial cardiac assessment.

#### Tier three: definitive bedside imaging with transesophageal echocardiography

For hemodynamically stable patients with persistent high suspicion, rescue transesophageal echocardiography (TEE) offers near-definitive bedside imaging (28). Performed by a trained operator, TEE provides high-resolution views of the thoracic aorta due to the probe's proximity to cardiac structures (29). It can clearly delineate the intimal flap, differentiate true and false lumens, identify entry and re-entry tears, and assess aortic valve function with doppler (29, 30). This level of anatomic detail approaches the diagnostic quality of gold-standard imaging and directly informs definitive management planning (Figure 1).

### 2.2 Core clinical value of the multimodal PoCUS approach

The systematic application of multimodal PoCUS provides significant clinical utility across four principal domains. First, its temporal efficiency accelerates the diagnostic pathway; a comprehensive bedside assessment can be completed within fifteen minutes (31, 32), substantially reducing the conventional “suspicion-to-decision” timeframe and preserving critical time for definitive surgical management (33). Second, it enhances patient safety by enabling evaluation at the point of care, thereby eliminating the hazards associated with transporting hemodynamically unstable patients (34). Additionally, as a non-ionizing and contrast-free modality, PoCUS offers a superior safety profile for individuals with renal impairment or other comorbidities (35, 36). Third, it facilitates diagnostic differentiation by concurrently screening for alternative life-threatening conditions that present similarly to ATAAD, such as acute coronary syndromes, massive pulmonary embolism, or cardiac tamponade, enabling efficient triage in time-sensitive settings (37, 38). Finally, it provides direct decision support by generating actionable findings. Positive results can immediately initiate surgical referral, guide resuscitation logistics, and optimize hemodynamic therapy (39–41), while negative or alternative findings allow for the timely redirection of the clinical workup, thereby conserving resources and streamlining patient care (42).

## 3 Challenges in routine implementation

While multimodal PoCUS holds significant promise for ATAAD screening, its evolution into a reliable, routine clinical tool is constrained by persistent technical, operational, and systemic barriers.

### 3.1 Technical and image acquisition limitations

Diagnostic utility is significantly influenced by patient-specific factors and the clinical environment. Suboptimal acoustic windows in patients with obesity, chronic lung disease, or on mechanical ventilation can obscure critical views (43, 44). The often chaotic emergency setting, combined with a patient's limited ability to cooperate with positioning, further impedes the acquisition of interpretable images (45). Specialized non-standard views, such as the suprasternal notch, are technically demanding, where operator skill directly determines both successful acquisition and accurate interpretation (46, 47). This inherent variability in image quality represents a fundamental limitation of PoCUS as a primary screening modality.

### 3.2 Operator dependence and training deficits

The diagnostic accuracy of this approach is inherently operator-dependent. The correct identification of subtle signs—such as a mobile intimal flap or the hemodynamic differentiation between true and false lumens—requires expertise that extends beyond standard focused cardiac ultrasound (48, 49). Current training paradigms for acute care providers predominantly emphasize assessments of cardiac function and volume status (50). Comprehensive instruction in systematic aortic evaluation, encompassing standardized scanning protocols, pathology recognition, and artifact discrimination, remains insufficient or unavailable in many settings (48, 51). This widespread training deficit constitutes a major human factor barrier to consistent, high-quality implementation.

### 3.3 Diagnostic performance and interpretive pitfalls

A clear understanding of PoCUS's diagnostic boundaries is essential. Its sensitivity, particularly for dissections confined to the aortic arch or distal ascending aorta, is inferior to that of CTA (52, 53). Consequently, a negative PoCUS examination cannot definitively exclude ATAAD, and clinicians must guard against the risk of premature diagnostic closure (53, 54). A central challenge lies in establishing unambiguous clinical protocols that position PoCUS as a rapid risk-stratification tool, not a definitive diagnostic test, thereby mandating and guiding appropriate confirmatory imaging (39, 53).

### 3.4 Integration into clinical workflow

Embedding multimodal PoCUS into emergency practice necessitates systematic workflow redesign. A primary obstacle is its formal integration into existing chest pain and back pain clinical pathways, which requires specifying precise indications, a standardized examination sequence, and clear interpretation guidelines (55). Furthermore, effective multidisciplinary collaboration must be established, defining explicit communication channels and decision-making responsibilities between the PoCUS operator, radiology, and cardiac surgery teams to prevent care delays and ensure seamless patient transition (56, 57).

### 3.5 Resource allocation and cost

Widespread institutional adoption demands sustained investment. This includes the procurement and maintenance of an adequate number of high-quality portable ultrasound systems accessible in key acute care areas (58, 59). More fundamentally, it requires establishing a robust training infrastructure involving simulation-based learning, credentialing processes, and ongoing competency assessment, representing a significant long-term commitment of financial resources and administrative support (60, 61).

## 4 Future directions: an integrated and intelligent screening strategy

To overcome existing barriers and maximize the clinical impact of multimodal PoCUS in ATAAD screening, future initiatives should aim to develop an integrated strategy combining technological innovation, standardized training, optimized clinical pathways, and multidisciplinary collaboration. This forward-looking framework systematically addresses current limitations.

### 4.1 Technological innovation: advancing image acquisition and interpretation

Future advancements will depend on the strategic convergence of artificial intelligence (AI) with ultrasound technology. AI-assisted tools can enhance real-time image quality, particularly for technically difficult examinations (62), and help clinicians by automating the acquisition of standard diagnostic views. Critically, advanced machine learning algorithms are under development for real-time diagnostic support, capable of automatically detecting key pathology such as an intimal flap, performing precise aortic measurements, and differentiating true from false lumen flow patterns (63, 64). These functions provide objective, quantitative data, thereby reducing interpretive subjectivity (65). Furthermore, the ongoing miniaturization and improved connectivity of ultrasound devices will facilitate direct integration with electronic health records, ensuring prompt data availability for clinical decision support (66).

### 4.2 Standardized training and competency assessment

Establishing a standardized training curriculum is essential for ensuring reliable application. This necessitates dedicated training and certification in focused aortic ultrasound for acute care providers (67). Such a program must emphasize hands-on simulation and be validated through competency-based assessments to guarantee that certified clinicians possess the requisite skills for accurate image acquisition and interpretation (48, 68).

### 4.3 Integration into evidence-based clinical pathways

For the technology to realize its potential, it must be formally embedded within clear clinical protocols. This requires the development of evidence-based, standardized algorithms that define the specific role of PoCUS in the evaluation of suspected ATAAD (69). These protocols must specify precise indications, a recommended examination sequence, and, most importantly, mandated subsequent actions based on the findings (70). For instance, a positive or equivocal result should trigger immediate confirmatory imaging or surgical consultation, while a negative result in a high-risk patient would still necessitate further investigation (71). This structured approach integrates PoCUS into a definitive diagnostic and treatment pathway, preventing delays in care.

### 4.4 Strengthening multidisciplinary care systems

The full value of this approach is realized through enhanced system-wide collaboration. An optimal model is an institutional fast-track pathway that directly links emergency PoCUS screening to rapid cardiology or radiology confirmation and expedited surgical evaluation (72). Concurrently, expanding tele-ultrasound networks can provide remote expert guidance for clinicians in community or resource-limited settings (73, 74). This regionalized strategy facilitates early triage and improves the efficiency of patient transfer to specialized centers, thereby enhancing outcomes across a broader healthcare network (75).

## 5 Summary

In summary, multimodal PoCUS represents a valuable bedside tool for the early screening of suspected ATAAD. Its principal advantage lies in providing rapid diagnostic information without the need for patient transfer, thereby expediting critical management decisions and potentially improving outcomes. It is essential, however, to correctly position PoCUS within the diagnostic pathway: it currently functions most effectively as a rapid screening and triage modality to identify high-risk patients requiring definitive imaging, not as a replacement for gold-standard tests like CTA. Addressing its current limitations necessitates a multifaceted strategy centered on technological innovation, standardized training, and systematic clinical integration. Future advancements in artificial intelligence, validated training programs, and structured clinical protocols promise to embed PoCUS more effectively within emergency care systems for acute aortic syndromes. This integration would directly support the overarching goals of early detection, timely referral, and precise intervention.

## Data Availability

The original contributions presented in the study are included in the article/Supplementary Material, further inquiries can be directed to the corresponding author.
